# Effect of 12-week rehearsal on cardiorespiratory fitness and body composition in Brazilian samba dancers

**DOI:** 10.31744/einstein_journal/2023AO0321

**Published:** 2023-12-06

**Authors:** Cicera Claudinea Duarte, Paulo Roberto Santos-Silva, Ana Carolina Paludo, Marcus Vinicius Grecco, Julia Maria D´Andrea Greve

**Affiliations:** 1 Department of Orthopedics and Traumatology Faculdade de Medicina Universidade de São Paulo São Paulo SP Brazil Movement Studies Laboratory, Department of Orthopedics and Traumatology , Faculdade de Medicina , Universidade de São Paulo , São Paulo , SP , Brazil .; 2 Faculty of Sports Studies Masaryk University Brno Czech Republic Faculty of Sports Studies , Masaryk University , Brno , Czech Republic .

**Keywords:** Body composition, Dancing, Exercise, Oxygen consumption, Adipose tissue

## Abstract

**Objective:**

To investigate the effect of 12 weeks of rehearsals on cardiorespiratory parameters and body composition in Brazilian samba dancers belonging to a first-league samba school.

**Methods:**

Twenty-six women were divided into a Samba Group (n=13) and a Control Group (n=13). Cardiorespiratory parameters (cardiopulmonary exercise test) and body composition (skinfold assessment) were assessed before and after the 12 weeks of rehearsals. The Samba Group rehearsed three times per week for 30–60 minutes, and the Control Group participated in no physical activity. A comparison test was performed within and between groups, with p<0.05 indicating statistical significance.

**Results:**

Compared with the Control Group, the Samba Group showed a significant increase in maximal oxygen uptake (19%), oxygen pulse (13%), and lean body mass (3%) and a decrease in body fat percentage (11%) and fat mass (12%).

**Conclusion:**

Twelve weeks of samba dance rehearsals improved the cardiorespiratory and body composition parameters in women dancers compared with the Control Group. These findings suggest that dancing samba regularly can increase physical activity levels and positively affect the health parameters of samba dancers.

## Summary

In Brief

Physical activity is highly recommended because of its health benefits, and samba dance is an example of an intense form of exercise. Duarte et al. demonstrated an improvement in healthrelated cardiorespiratory and body composition parameters in Brazilian samba dancers during 12 weeks of rehearsals before the well-known carnival, compared with a Control Group.

## Abstract (key points)

Highlights

Samba rehearsals can increase cardiorespiratory function in dancers up to 19% of VO2max.

Samba rehearsals can decrease up to 1.9kg of fat mass and increase 1.3kg of lean body mass.

In a samba rehearsal session, dancers spend 50% of their time at an intensity above 83% of the maximum.

## Graphical abstract

**Figure:**
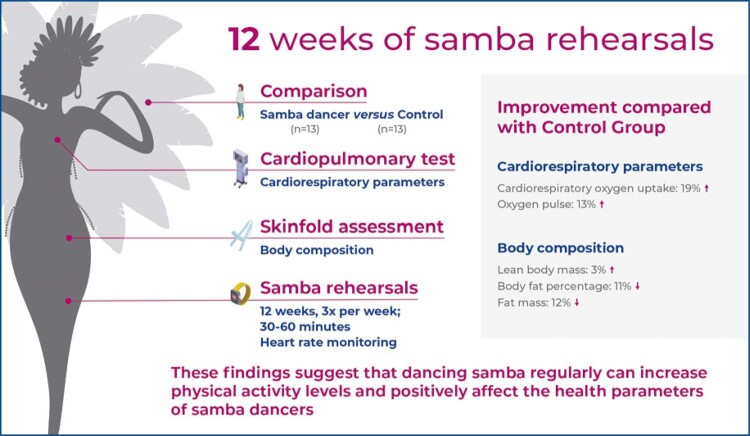

## INTRODUCTION

Participating in dance programs requires the ability of muscles to use energy and generate work to perform choreography-related movements. ^( 1 )^ Because of the energy required, dance programs have been used as an exercise modality to improve functional, metabolic, and cardiovascular parameters. ^( 2 )^ Additionally, dance programs have shown positive changes in body composition by increasing muscle mass and decreasing body fat mass. ^( 3 , 4 )^ Among dance styles, the “samba dance” requires the dancer to have a certain fitness level. The physical efforts during a 40-minute dance session can raise the heart rate (HR) by approximately 60–90% of the maximum, have an energy expenditure of 9.9kcal/min, and a metabolic equivalent of 13.5 (METs). This form of dance is considered moderate-vigorous intensity exercise, as demonstrated in previous study elsewhere. ^( 5 )^

Dancers belonging to samba schools competing in the Carnival Parade play a specific role. The dancers perform the samba rhythm individually, going through the “floor competition” for 17 minutes. Before the competition, the samba schools organize rehearsals to familiarize the dancers with the samba rhythm and choreography. Rehearsals can be highly demanding, especially when approaching the competition.

Therefore, if samba is considered an intense exercise, then we can assume that dancers who regularly participate in the samba school can change their physical fitness and body composition during the rehearsal period. However, the magnitudes of these changes have not yet been investigated. This study aimed to evaluate the effect of 12 weeks of rehearsals on the cardiorespiratory parameters and body composition in samba dancers belonging to a first-league samba school in São Paulo, Brazil. We hypothesized that the rehearsal sessions would be high-intensity, and after 12 weeks, these sessions would significantly improve the cardiorespiratory parameters and body composition in samba dancers compared with non-physically active participants.

## OBJECTIVE

To investigate the effect of 12 weeks of rehearsals on cardiorespiratory parameters and body composition in Brazilian samba dancers belonging to a first-league samba school.

## METHODS

### Participants

Twenty-six women participated in this study and were separated into the Samba Group (SG, n=13) and the Control Group (CG, n=13), consisting of non-active women. Samba dancers from the same samba school, preparing to compete in the Carnival Parade in São Paulo, Brazil, were eligible for our study group. The inclusion criteria for dancers were as follows: minimum 1-year experience in the selected samba school, not performing samba dance for at least 6 months before the study started, participation in the samba school rehearsals during the study period (12 weeks), and signing the ethical consent form. The exclusion criterion was missing 10% of the rehearsal sessions. The inclusion criteria of the CG included women with similar cardiorespiratory and body composition as the SG, being sedentary for 6 months or more (no regular physical activity), ^( 6 )^ not being a professional or recreational samba dancer at least 1 year before the study, and signing the ethical consent form.

Participants of both groups also met the criteria: no history of chronic systemic disease or locomotion; age between 20 and 40 years; body mass index less than 30kg ^.^ m ^-2^ ; no pregnancy, no smoking, no regular physical activity for at least 6 months; and no participation or intent to participate in any diets during the study period. The exclusion criterion for both groups was the presence of abnormal hemodynamic responses during cardiopulmonary exercise tests.

This study was approved by the local Research Ethics Committee of the Faculty of Medicine of the *Universidade de São Paulo* (CAAE: 06311912.1.0000.0065; # 91.729), and informed consent was obtained from each participant before the commencement of the study.

### Study design

This study comprised three phases. The first phase involved the selection of a samba school along with an invitation for samba dancers to participate and the recruitment of women to the CG. After selection, the participants in both groups underwent a cardiorespiratory test and body composition assessment. In the second phase, the SG underwent 12 weeks of rehearsals in the selected samba school, and the CG went about their everyday activities. In the third phase, after 12 weeks, all participants underwent the same cardiorespiratory test protocol and body composition assessment.

### Procedures

#### Cardiorespiratory fitness

Cardiorespiratory fitness was evaluated using the progressive maximal cardiopulmonary exercise test. Before the test, the participants received an explanation of the test protocol. ^( 7 )^ The maximal test was performed on a motor-driven belt treadmill (h/p/cosmos ^®^ sports & medical gmbh, pulsar, Nussdorf-Traunstein, Germany) with speed (km ^.^ h ^-1^ ) and gradient (%) variables. The modified Heck protocol was used with fixed speed and increasing slope increments (ramp style) of 2% every minute. ^( 8 )^ Before starting the test, both groups were tested at different speeds per the ramp protocol (4.8, 6.0, 6.5, and 7.2 km ^.^ h ^-1^ ), and the most comfortable speed was chosen individually (self-chosen protocol). A 12-lead electrocardiogram (HeartWare ^®^ , Ergo 13, Belo Horizonte, Brazil) was used to continuously monitor all tests at rest, during exercise testing, and recovery. The sequence of monitoring the electrocardiogram was used as described earlier. The HR was recorded at 60-second intervals during the exercise and recovery phases. Blood pressure was monitored as described above while the subjects were at rest and during minutes 1, 3, and 6 after recovery.

The test protocol lasted 8–15 minutes. ^( 9 )^ During the test, the perceived exertion was recorded at each stage of the velocity increment using the 6–20 Borg Scale. ^( 10 )^ Oxygen consumption was also monitored during the test by continuous gas exchange, breath-by-breath, obtained using a computerized metabolic analyzer (CPX/Ultima, Medical Graphics ^®^ , St. Paul, MN, USA) with direct oxygen uptake measurement, carbon dioxide production, and pulmonary ventilation. The flow meters and gas analyzers were calibrated before and after each test with a known volume in the syringe (3L) and known gas mixtures using a three-point measure: a calibration gas (CO _2_ 4.96%, O _2_ 20.9%, and N _2_ 12.1% balance) and a reference gas (room air at ambient temperature and pressure, saturated to standard temperature and pressure, dry) (Messer Special Gases, Inc.). To determine the maximal oxygen uptake (VO _2_ max), at least three of the following criteria were considered: a plateau in oxygen consumption with an increasing workload between the penultimate and last stages of the exercise test ≤2.1 mL·kg ^-1^ ·min ^-1^ , ^( 11 )^ a respiratory exchange ratio >1.10, ^( 12 )^ HR ≥95% of the age-predicted maximum HR by the Tanaka equation, ^( 13 )^ and a perceived exertion >18. ^( 14 )^

#### Body composition

Total body mass was measured on a mechanical scale (Filizola ^®^ Instruments, Brazil) with 0.1kg precision. The height was determined using a stadiometer with 0.1cm precision. ^( 15 )^ A scientific adipometer (Lange ^®^ , Cambridge Scientific Industries, Inc., Cambridge, MD, USA) estimated body fat by measuring the thickness of the skin folds using the seven folds protocol (in the pectoral, midaxillary, triceps, subscapularis, abdominal, thigh, and suprailiac areas). ^( 16 , 17 )^

Body density and body fat percentage were estimated by the following equations: EQUATION A:Body Density = (1,097- (0.0004697 x Σ seven skinfolds) + (0.00000056 x (Σ seven folds)_2_ - (0.00012828 x age). Seven skinfolds: pectoral, abdominal, thigh, triceps, suprailiac, subscapularis, and midaxillary. ^( 18 )^ EQUATION B:Body Fat % = (4.95/body density) - 4.5) x 100. ^( 19 )^

#### Samba dance rehearsals

The rehearsals occurred at the selected samba school location over 12 weeks, three times per week, lasting 30–60 minutes. Each rehearsal was divided into three phases: warm-up, main rehearsal, and dispersion (closure). During the warm-up, dancers performed the samba while playing music at a slow rhythm. The main rehearsal consisted of performing samba dance with the official music while increasing rhythm and including movements related to the choreography. Dispersion comprises the final part, in which the dancer needs to accelerate their movements to finish the parade on time. The intensity of each phase was monitored using mean HR values. Dancers used an HR monitor (Polar ^®^ Team System, T31, Kempele, Finland), and every 5 minutes, the HR was recorded. Rehearsals lasting <30 minutes were discarded because they were carried out in the rain or in environmental conditions that did not allow the researcher to monitor the HR ( *e.g* ., a place with too many people, specific samba clothing that did not allow the dancer to wear the belt, or when monitors were inaccessible to dancers). Data from seven of 37 sessions were excluded. The rehearsal intensity was estimated by considering the percentage of HRmax from VO _2_ max using the linear regression equation %HRmax = 0.64 × %VO^2^max + 37. ^( 20 )^

## Statistical analysis

The normal distribution of the data was verified using the Shapiro–Wilk normality test. After the assumption of homogeneity and sphericity were confirmed, intra and intergroup comparisons were tested using repeated-measures two-way analysis of variance ( *e.g* ., SG pre-post, CG pre-post, SG post *versus* CG post, and SG post *versus* CG pre). The Bonferroni post hoc test was used to detect significant differences. Additionally, the effect sizes (ES) were performed only in the SG to determine the significance of the difference between pre- and post-rehearsal values with a 95% confidence interval (95%CI). ES was calculated considering the Cohen’s d value, corrected for bias using the Hedges formula, and following the qualitative classification: <0.2 trivial; 0.2 to <0.5 small; 0.5 to <0.8 medium; ≥0.8 large. ^( 21 )^ Sigma Stat software (Sigma Stat 3.5, Systat Software Inc, Ashburn, VA, USA) was used to perform all analyses, with a p-value of <0.05 indicating statistical significance.

## RESULTS

Table 1 presents the descriptive characteristics, cardiorespiratory parameters, and body composition of both groups before and after the 12 weeks of rehearsals. No significant differences were found in the baseline measures for all parameters ( *e.g* ., cardiorespiratory and body composition) between the groups, assuming similarities among participants. Samba dancers showed a significant increase of 19% in VO _2_ max and 13% in peak oxygen pulse after 12 weeks of rehearsals compared with the CG, demonstrating a large effect of the intervention (ES=1.86 and 1.03, respectively). Similarly, a significant decrease of 1.9kg in fat mass, 11% in fat percentage, and an increase of 1.3kg in lean body mass after 12-week rehearsals compared with the CG demonstrated a medium effect of the intervention (ES=0.43; 0.54 and 0.46, respectively). No significant changes were observed in any of the remaining variables.

**Table 1:** Cardiorespiratory parameters and body composition in the Samba Group and Control Group

Variables	Baseline (pre)	After 12 weeks (post)	p value (pre-post)	ES Cohen’s d (95%CI)
Age (years)				
SG	29±4			
CG	27±3			
VO _2_ max (mL·kg ^-1^ ·min ^-1^ )				
SG	31.2±2.7	37±3.5 ^† ‡ §^	0.001	1.86 (-2.71 – -0.89)
CG	29.8±4.3	30.5±3.5	0.360	
PO _2_ (mL ^.^ HR ^-1^ )				
SG	10.1±1.4	11.4±1.1 ^† ‡ §^	0.001	1.03 (-1.82 – -0.18)
CG	8.5±1.1	8.9±0.7	0.080	
BM (kg)				
SG	60.3±6.1	59.5±6.2	0.136	0.13 (-0.64–0.90)
CG	56.6±7.9	56.4±7.9	0.759	
Height (m)				
SG	1.63±0.5			
CG	1.60±0.5			
BMI (kg ^.^ m ^-2^ )				
SG	22.8±1.9	22.2±2.4	0.134	0.28 (-0.50–1.04)
CG	22±2.7	21.9±2.4	0.722	
LBM (kg)				
SG	44.2±2.7	45.5±2.9 ^† ‡ §^	0.004	0.46 (-1.23–0.33)
CG	40.9±4.4	40.4±3.9	0.401	
BF (%)				
SG	26±5.6	23.1±5.2 ^†^	0.001	0.54 (-0.26–1.30)
CG	27.4±5.7	27.7±5.7	0.874	
FM (kg)				
SG	15.9±4.6	14.0±4.2 ^†^	0.001	0.43 (-0.36–1.19)
CG	15.8±5.4	16.0±5.2	0.928	

Intragroup analysis ^†^ p<0.05; Intergroup analysis; ^‡^ p<0.05 for SG post *versus* CG post; ^§^ p<0.05 for SG post *versus* CG pre p<0.05.

SG: samba Group CG: control group; VO _2_ max: maximal oxygen uptake (mL·kg ^-1·^ min ^-1^ ); PO _2:_ oxygen pulse (mL ^.^ HR ^-1^ ); BM: body mass; BF: body fat; FM: fat mass; LBM: lean body mass; BMI: body mass index.

Table 2 shows the intensity of the samba rehearsals, which were separated into three phases (warm-up, main rehearsal, and dispersion). The samba dancer spent 8% in phase 1 (61% VO _2_ max), 42% in phase 2 (62%–70% VO _2_ max), and 50% in phase 3 (72% VO _2_ max). In phase 3, an elevated intensity was demonstrated by an HR above 83% of the maximum, corresponding to 72% of VO _2_ max.

**Table 2:** Rehearsal intensity by phase in Samba Group (n=13)

Rehearsal phase	% HRmax	%VO _2_ max
Phase 1 (warm-up)	56–76	30–61
Phase 2 (main part)	77–82	62–70
Phase 3 (dispersion)	83	72

%HRmax: percentage of maximal heart rate; %VO _2_ max: percentage of maximal oxygen uptake.

## DISCUSSION

The present study aimed to investigate the effects of 12 weeks of rehearsals on cardiorespiratory parameters and body composition in Brazilian samba dancers. The samba dancers presented a significant increase in maximal oxygen uptake, oxygen pulse, lean body mass, and decreased body fat percentage and fat mass after the rehearsal period, compared with the CG.

As hypothesized, the samba rehearsal was high-intensity. The samba dancers demonstrated metabolic consumption of VO _2_ with values between 42% and 72% of the VO _2_ max, or 56% and 83% of the HRmax, representing light to vigorous intensity. ^( 20 )^ A previous study conducted in our laboratory was the first to monitor the metabolic response to samba dance for 40 minutes in dancers presenting HR values at approximately 60–90% of the maximum, an energy expenditure of 9.9kcal/min, and a metabolic equivalent of 13.5. ^( 5 )^ Samba rehearsals are an important part of the samba school, as participants become familiar with the rhythm and choreography that will be performed in the Carnival Parade. Based on the aforementioned results, samba dance has an important aerobic characteristic as an exercise using oxygen in the metabolic or energy-generating processes of the body. ^( 22 )^ Thus, samba rehearsals can be an exercise option for adults to meet the American College of Sports Medicine recommendations for physical activity, which suggest performing 3–5 days a week of continuous or intermittent aerobic activity over moderate to vigorous intensity, varying from 20–60 minutes. ^( 7 )^

The study also confirmed the positive effect of samba rehearsal sessions on improving VO _2_ max by 19%, with a large effect. Discussing the VO _2_ max parameter in samba dancers is restricted, as there are minimal studies on this modality; therefore, we rely on comparisons with other dance styles. In Zumba, dancers demonstrated an improvement of 10% in VO _2_ max after 12 weeks of aerobic exercise or Zumba dance. ^( 23 )^ Participants in aerobic dance programs also showed an increase in VO _2_ max ranging from 7%–10%. ^( 22 , 24 )^ Dance can be viewed as having a similar approach to interval training where variations in intensity can favorably improve various cardiovascular parameters. ^( 25 )^ As dance is generally perceived to be joyful, its adoption favors more regular participation throughout life. ^( 26 )^

Moreover, cardiovascular parameters, such as the oxygen pulse, also improved in samba dancers with a large effect. The curve rises hyperbolically and consistently to near-maximal effort. This reflects the myocardial oxygen supply and cardiac functional reserve under physiological stress: it increases linearly until it approaches the maximum value. ^( 27 , 28 )^ The oxygen pulse, which depends on the volume of O _2_ extracted by peripheral tissue and captured in the pulmonary circulation during each HR, showed a 13% increase in samba dancers after the rehearsal period. Samba dancers can regularly increase the stroke volume and peripheral oxygen uptake at maximum effort intensity. This can be highly beneficial for improving heart health and muscle performance owing to the increased peripheral oxygen extraction. ^( 27 , 28 )^

Improvements in cardiorespiratory capacity trigger positive changes in body composition. ^( 29 )^ The samba dancers in this study also showed improved body composition, increased lean body mass, and decreased body fat percentage and fat mass. These changes were possibly due to higher energy expenditure after exercise and maintaining a high resting metabolic rate for long periods. ^( 30 )^ These findings agree with those of previous studies on dancers that also demonstrated a reduction in body fat. ^( 30 - 32 )^ Of note, the samba dancers in the study were not involved in a diet program or any food restrictions during the 12-week rehearsal period. Therefore, it is possible to speculate that samba dance practices can reduce body fat and be used as a health promoter, being more attractive and accessible owing to its cultural and recreational aspects. ^( 30 )^

Although the current study provides novel information describing the positive effects of rehearsals on samba dancers, some limitations should be considered. The selection of only one samba school and the inherently small sample size limit the generalizability of the findings. Samba rehearsals are linked to the samba rhythm called “ *samba enredo* .” Samba schools practice different *samba enredo* with different intensities, resulting in varying effects on cardiorespiratory parameters and body composition. Additionally, HR measurements during the rehearsals and body composition evaluations were not performed with the standard tools, such as the cardiorespiratory parameters. Therefore, we know that these indirect methods will present some assessment errors, resulting in overestimation or underestimation of the actual results. Further studies are needed to consider these limitations and improve our knowledge.

### Practical and clinical implications

Samba sessions can be a beneficial strategy for improving health-related components in adult women and protecting them against chronic non-communicable diseases. As samba is a popular dance in Brazil, it can be a positive opportunity to be introduced and recommended in health policy as a preventive health practice to improve cardiorespiratory fitness and body composition. For samba dancers specifically, the positive effect of rehearsals is promising and can help improve their fitness level and, consequently, their performance in the Carnival Parade.

## CONCLUSION

Based on the results, this study demonstrates that 12 weeks of samba rehearsals improve cardiorespiratory functional fitness and body composition parameters in samba dancers compared with the Control Group. Rehearsals significantly affected cardiorespiratory parameters (large effect) more than body composition (small effect). The impact of rehearsals can be fundamental for improving the fitness level of samba dancers, resulting in better performances in carnival competitions. Moreover, samba dance is characterized as high-intensity exercise, which can be considered an alternative to improve health-related physical fitness as it is performed with joy and humor. We recommend this methodology for future research. For sedentary individuals, the fitness level should be considered because samba is high-intensity; therefore, these findings should be generalized with caution.
